# YAP/TAZ-associated cell signaling – at the crossroads of cancer and neurodevelopmental disorders

**DOI:** 10.3389/fcell.2025.1522705

**Published:** 2025-01-28

**Authors:** Aderonke O. Ajongbolo, Sigrid A. Langhans

**Affiliations:** ^1^ Division of Neurology and Nemours Biomedical Research, Nemours Children’s Health, Wilmington, DE, United States; ^2^ Biological Sciences Graduate Program, University of Delaware, Newark, DE, United States

**Keywords:** Hippo, brain, cancer, neurodevelopmental disorder, microenvironment, YAP/TAZ signaling

## Abstract

YAP/TAZ (Yes-associated protein/paralog transcriptional co-activator with PDZ-binding domain) are transcriptional cofactors that are the key and major downstream effectors of the Hippo signaling pathway. Both are known to play a crucial role in defining cellular outcomes, including cell differentiation, cell proliferation, and apoptosis. Aside from the canonical Hippo signaling cascade with the key components MST1/2 (mammalian STE20-like kinase 1/2), SAV1 (Salvador homologue 1), MOB1A/B (Mps one binder kinase activator 1A/B) and LATS1/2 (large tumor suppressor kinase 1/2) upstream of YAP/TAZ, YAP/TAZ activation is also influenced by numerous other signaling pathways. Such non-canonical regulation of YAP/TAZ includes well-known growth factor signaling pathways such as the epidermal growth factor receptor (EGFR)/ErbB family, Notch, and Wnt signaling as well as cell-cell adhesion, cell-matrix interactions and mechanical cues from a cell’s microenvironment. This puts YAP/TAZ at the center of a complex signaling network capable of regulating developmental processes and tissue regeneration. On the other hand, dysregulation of YAP/TAZ signaling has been implicated in numerous diseases including various cancers and neurodevelopmental disorders. Indeed, in recent years, parallels between cancer development and neurodevelopmental disorders have become apparent with YAP/TAZ signaling being one of these pathways. This review discusses the role of YAP/TAZ in brain development, cancer and neurodevelopmental disorders with a special focus on the interconnection in the role of YAP/TAZ in these different conditions.

## Introduction

Since the discovery of the Hippo gene in *Drosophila* in the early 2000s (Tapon et al., 2002; Kango-Singh et al., 2002; Harvey et al., 2003; Udan et al., 2003; Pantalacci et al., 2003; Kim and Jho, 2018), intense investigations have revealed a role for the Hippo signaling pathway in modulating organ size regulation, tissue homeostasis, promoting stem cell differentiation, and cancer progression (Piccolo et al., 2013; Piccolo et al., 2023; Zheng and Pan, 2019; Chen et al., 2019; Driskill and Pan, 2023). In more recent years, research in genetics has identified Yes-associated protein (YAP) and paralog transcriptional co-activator with PDZ-binding domain (TAZ) as the key and major downstream effectors of the Hippo pathway (Ma et al., 2019), a signaling cascade that is evolutionarily preserved and controls numerous biological processes such as cellular proliferation, regulation of organ sizes, programmed cell death, and tissue regeneration. YAP/TAZ are transcriptional cofactors that play a crucial role in defining cellular outcomes, including differentiation, proliferation, and apoptosis. Given their significance, YAP/TAZ regulate a wide array of physiological cellular mechanisms, positioning them as key factors in upholding tissue equilibrium and as potential therapeutic targets across various pathological contexts (Piccolo et al., 2023; Zarka et al., 2021; Dey et al., 2020). YAP/TAZ are not solely under the regulation of the Hippo pathway core kinases; additionally, they intercommunicate with various other signaling pathways including EGFR, WNT, TGF-β, and Notch, all of which play a role in processes related to development and cell proliferation (Lo Sardo et al., 2018; Zinatizadeh et al., 2021; Zhong et al., 2024). The mechanical forces generated from cell–cell contacts, cell–extracellular matrix (ECM) interactions, and the tissue microenvironment have been recognized to also activate the YAP/TAZ transcriptional effector which further regulate gene transcription and thus coordinate cells during growth, proliferation, morphogenesis, migration, and cell death (Bissell and Barcellos-Hoff, 1987; Bissell and Aggeler, 1987; Zhu et al., 2020; Liu et al., 2015; Miller and Sewell-Loftin, 2021; Cai et al., 2021; Dupont et al., 2011). Numerous studies have demonstrated the involvement of this pathway in the normal brain development, spanning the formation of the neural tube to the maturation and enhancement of the cerebral cortex, cerebellum, and the ventricular system (Terry and Kim, 2022; Ouyang et al., 2020; Sahu and Mondal, 2021). The aberration of this pathway is prevalent in a variety of human malignancies where the essential nature of YAP/TAZ in regulating several key features of cancer has been widely observed. An extensive analysis of 9,125 tumor specimens unveiled a widespread dysregulation of the co-transcription factors YAP/TAZ across diverse cancer categories including medulloblastoma, glioma, neuroblastoma, colorectal, liver, lung, and pancreatic cancers (Sanchez-Vega et al., 2018). Recently, new studies have emerged implicating YAP/TAZ in neurodevelopmental disorders and defective neurogenesis which will be discussed later in this review. Findings from these studies suggest that the dysregulation of the YAP/TAZ signaling pathways may have a significant impact on the development of these disorders (Sahu and Mondal, 2021; Nussinov et al., 2023; Jin et al., 2020). Understanding the precise mechanisms by which YAP/TAZ contribute to neurodevelopmental disorders could provide new insights into their etiology and open potential avenues for therapeutic intervention.

## Overview of the Hippo-YAP/TAZ signaling pathway

**FIGURE 1:**
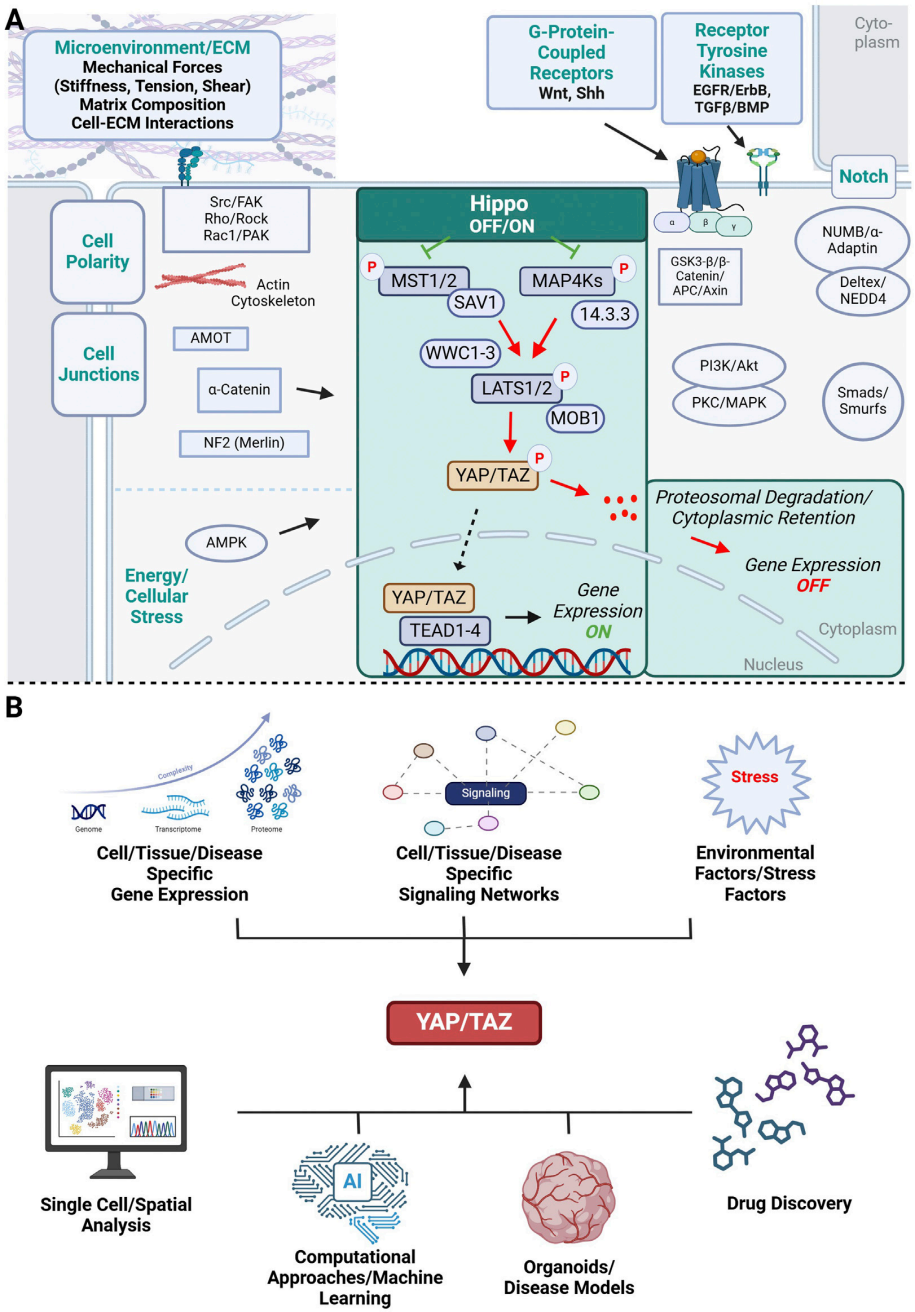
Schematic representation of the YAP/TAZ signaling network. **(A)** Microenvironmental factors and cellular signaling networks that influence YAP/TAZ signaling and have been implicated in brain development, neurodevelopmental disorders and cancer. Highlighted in green is the canonical Hippo-YAP/TAZ-TEAD signaling cascade. Non-canonical YAP/TAZ activation is influenced by adjacent cells (cell polarity complexes, cell junctions, Notch signaling), microenvironmental factors such as mechanical forces (stiffness, tension, shear stress) and extracellular matrix (ECM) composition, and soluble signaling molecules triggering receptor tyrosine kinase (EGFR, TGF-β) and G-protein coupled receptor signaling pathways (Wnt, Shh). All these factors have been shown to influence normal brain development, can be dysregulated in neurodevelopmental disorders and promote cancer progression. Common intracellular signaling molecules relating extracellular factors to downstream YAP/TAZ activation are shown. **(B)** YAP/TAZ signaling is a complex signaling network that is controlled by cell- and tissue specific gene expression signaling networks and environmental factors beyond the cellular/tissue level contribute to the complex regulation of YAP/TAZ signaling. The complexity of this network will require multiplexed approaches when targeting YAP/TAZ in drug discovery including large-scale omics approaches that need to be integrated with representative disease models and computational methods for successful drug development. Illustration created in https://BioRender.com.

The Hippo pathway consists of a core kinase, effectors, cofactors, and transcription factors and is highly conserved in mammals (Hong et al., 2016). Cell contact, cell polarity, as well as metabolic and mechanical signals, undergo alterations throughout organ development and growth in order to effectively coordinate these highly complex processes, thereby regulating the function of the core components of the Hippo pathway (Panciera et al., 2017; Meng et al., 2016; Santinon et al., 2016). The Hippo pathway functions as a kinase cascade within cellular signaling mechanisms. Numerous investigations have demonstrated the significance of the Hippo signaling pathway in maintaining tissue homeostasis, promoting regeneration, and influencing the onset and progression of tumors (Dey et al., 2020; Dong et al., 2007). In mammals, activation of the canonical Hippo pathway results in the assembly of the sterile 20‐like protein kinase (MST1/2; mammalian homologs of Hippo kinase) and Salvador 1 (SAV1)/WW45 complex, leading to the subsequent phosphorylation of the large tumor suppressor (LATS1/2)/Warts (Wts) and Mps One Binder kinase activator 1 (MOB1). Following this, the activated LATS1/2‐MOB1 complex phosphorylates YAP/TAZ (Yorkie/Yki in *Drosophila*), causing their sequestration and breakdown in the cytoplasm. As a result, this mechanism prevents the accumulation of YAP/TAZ in the nucleus and the ensuing expression of downstream genes (Zhou and Zhao, 2018). The direct regulation of YAP/TAZ through LATS is defined as “canonical signaling”. This contrasts with the concept of “non-canonical signaling” used in recent studies to describe situations where the activity of YAP and TAZ is controlled independently of the LATS kinase. In addition to the canonical Hippo pathway, the MST1/2‐SAV1‐LATS1/2‐MOB1‐YAP/TAZ axis, other factors such as neurofibromin 2 (NF2) (Dong et al., 2007), mitogen‐activated protein kinase kinase kinase kinases (MAP4Ks) (Meng et al., 2015), the cytoskeleton, focal adhesions (Dupont et al., 2011) and nuclear Dbf2‐related1/2 (NDR1/2) (Meng et al., 2015) have been recognized as pathway regulators, thereby enhancing our understanding of the complexity of the Hippo signaling pathway (Figure 1).

In contrast, when the Hippo pathway is turned off, dephosphorylated YAP/TAZ translocate to the nucleus where they interact with other transcription factors to modulate the transcription of downstream genes (Driskill and Pan, 2021). YAP/TAZ does not directly bind DNA; hence, they induce their biological functions through the formation of complexes with a different transcription factor known as TEAD (Transcriptional enhanced associate domain) DNA-binding family members (TEAD1-4). The expression of downstream target genes associated with the Hippo pathway (Wang et al., 2018; Totaro et al., 2018a), such as connective tissue growth factor (CTGF), cysteine-rich angiogenic inducer 61 (CYR61), ankyrin repeat domain 1 (ANKRD1), and MYC proto-oncogene transcription factor (MYC), plays a crucial role in regulating cellular proliferation, differentiation, survival, migration and viability (Fu M. et al., 2022; Ehmer and Sage, 2016; Misra and Irvine, 2018). Dysregulation of the Hippo pathway occurs in a broad range of human carcinoma, including lung (Liang et al., 2024; Yoo et al., 2021), colorectal (Hong et al., 2016; Mouillet-Richard and Laurent-Puig, 2020), breast (Kyriazoglou et al., 2021), ovarian (Clark et al., 2022), pancreatic (Ansari et al., 2019), gastric (Seeneevassen et al., 2022), liver (Driskill and Pan, 2021) and brain (Ahmed et al., 2017; Masliantsev et al., 2021) cancer. It is important to note that YAP/TAZ undergo a continuous process of phosphorylation and dephosphorylation, although the mechanism governing the activity of the phosphatases is not well known (Driskill and Pan, 2021). Recent research conducted in both *Drosophila* and mammalian cells utilizing live cell tracking techniques has revealed the rapid movement of Yki/YAP between the cytoplasm and nucleus (Ege et al., 2018; Manning et al., 2018). Changes in the Hippo signaling pathway have been observed to impact the phosphorylation of YAP, consequently affecting the rates at which YAP enters and exits the nucleus. This dynamic transportation of YAP is facilitated by nuclear pore complexes that are sensitive to mechanical signals, enabling the process of nuclear import and export (Li et al., 2023).

## Other signaling pathways that activate and intercommunicate with YAP/TAZ

A cell functions as a dynamic entity where multiple processes take place concurrently. Specifically, the interaction between intra- and intercellular signaling pathways greatly influences various aspects of a cell’s biological processes such as its life cycle, differentiation, proliferation, growth, and regeneration, consequently affecting the normal operation of an entire organ. Communication between different signaling pathways plays a crucial role in modulating the key elements of the Hippo pathway and the localization of YAP/TAZ (Govorova et al., 2024). As mentioned earlier, YAP/TAZ can be activated and intercommunicate with other signaling pathways. The three major pathways that previous studies have shown to activate or intercommunicate with YAP/TAZ to regulate expression of downstream target genes involved in cell proliferation and apoptosis are the epidermal growth factor receptor (EGFR)/ErbB family, Notch, and Wnt signaling pathways.

### EGFR/ErbB family signaling

The epidermal growth factor receptor (EGFR) family of receptor tyrosine kinases (RTKs) consists of four members, EGFR/ErbB1/Her1, ErbB2/Her2, ErbB3/Her3 and ErbB4/Her4 and modulates an intricate signaling system involved in key cellular functions such as cell growth, cell division, cell migration, cell adhesion and apoptosis (Yarden and Pines, 2012; Citri and Yarden, 2006). Activation of the inherent kinase domain and phosphorylation on distinct tyrosine residues in the cytoplasmic tail are induced by the binding of ligands like EGF, amphiregulin, or HB (heparin binding)-EGF. The interconnection between the EGFR and Hippo-YAP/TAZ signaling pathways has been observed in a variety of conditions. For example, EGFR-dependent PI3K (phosphatidylinositol-3 kinase)-Akt signaling was discovered to serve as an upstream signal for the activation of YAP in the context of acute kidney injury (Chen et al., 2018) while ErbB2 drove YAP activation resulting in heart regeneration (Aharonov et al., 2020). Several groups described a link between EGFR signaling and the Hippo pathway in relation to the development of cancer, including in hepatocellular carcinoma (HCC) (Xia et al., 2018), lung cancer (Liang et al., 2024; Hsu et al., 2019) and breast cancer (Xu et al., 2024). The molecular links between EGFR and Hippo-YAP/TAZ signaling are varied. In head and neck squamous cell carcinoma (HNSCC) cells, one study demonstrated that the activation of EGFR results in the phosphorylation of one of the core Hippo pathway components, MOB1, which hinders the function of LATS1/2, consequently leading to the aberrant activation of YAP/TAZ independently of EGFR’s typical signaling targets, including PI3K (Ando et al., 2021). Zhang and Li (Zhang and Li, 2022) demonstrated that the activation of the EGFR pathway predominantly occurred through PI3K-PDK (pyruvate dehydrogenase kinase) 1 signaling, circumventing the conventional RhoA pathway in favor of the Akt pathway to induce YAP activation in proliferative vitreoretinopathy. This was also seen in hepatocellular carcinoma where it was shown that EGFR mainly acts via PI3K-PDK1 signaling to activate and regulate YAP (Xia et al., 2018) whereas in glioblastoma, YAP nuclear translocation was regulated by EGFR through activation of the PTEN (phosphatase and tensin homolog)/Akt axis (Masliantsev et al., 2023). Blocking EGFR activity with AG1478 or EGFR knockdown in cervical cancer cells resulted in the elimination of YAP-induced cell proliferation (He et al., 2015). The authors demonstrated that the Hippo pathway interacts with the ErbB signaling pathway, establishing positive feedback signaling loop that plays a crucial role in regulating cervical cancer progression. Notably, HPV16 E6 inhibits the proteasome-dependent degradation of YAP, thereby sustaining the levels of YAP protein in cervical cancer cells and potentially promoting cancer cell growth (He et al., 2015). While our group has described a link between EGFR signaling and YAP involving Na,K-ATPase β_2_-subunit/AMOG (adhesion molecule on glia) and neurofibromin-2/Merlin in cerebellar granule cells (Litan et al., 2019), little is known about the interaction of EGFR and YAP in neurological diseases. However, recent studies have implicated Neuregulin 1 with its receptor ErbB4 in neurodevelopmental, neurodegenerative and psychiatric disorders (Shi and Bergson, 2020; Perez-Garcia, 2015; Adashek et al., 2024) and this pathway has been shown to interact with Hippo/YAP signaling (Haskins et al., 2014; Yan et al., 2017; Sudol, 2014).

### Notch signaling

The Notch pathway is an evolutionary highly conserved signaling pathway that plays an important role in tissue and organ development. Unlike other well-known signaling pathways such as EGFR, Sonic hedgehog (Shh), Wnt, or BMP (bone morphogenetic protein)/TGF (transforming growth factor)-β signaling that mostly employ soluble ligands, Notch is activated through a transmembrane ligand/transmembrane receptor interaction of juxtaposed cells (Siebel and Lendahl, 2017; Zhou et al., 2022). Two primary modalities have been identified in the interaction between the YAP/TAZ and Notch signaling cascades: the regulation of Notch ligands or receptors by YAP/TAZ at the transcriptional level, and the co-regulation of common target genes by both YAP/TAZ and NICD (Notch intracellular domain) suggesting that there is a notable interplay between YAP/TAZ and the Notch signaling pathway (Totaro et al., 2018b).

There have only been a few studies that reported upstream regulation of YAP/TAZ activity via the Notch signaling pathway (Li et al., 2012; Slemmons et al., 2017; Lu et al., 2016). In murine neural stem cells, using gain- and loss-of-function experiments, findings indicated that the Notch signaling pathway exerts a positive upstream control on YAP activity, thereby regulating cell proliferation. Subsequent investigations unveiled that the RBPJ (recombination signal binding protein for immunoglobulin kappa J) transcription factor within the canonical Notch signaling pathway directly oversees the transcription of YAP1 protein by engaging its promoter sequence (Li et al., 2012). These investigators further noted that RBPJ could bind to TEAD2’s promoter sequence as well; nevertheless, this action alone was inadequate to initiate TEAD2 transcription. Correspondingly, similar outcomes were documented in research by Slemmons et al. (2017) on human rhabdomyosarcoma cells, employing gain- and loss-of-function methods to exhibit the upregulation of YAP activity by the Notch signaling pathway. Also, this study highlighted that the Notch signaling pathway may also facilitate YAP nuclear translocation. However, the underlying mechanism governing this process remains elusive, and it remains uncertain whether it involves the core Hippo signaling cascade (Slemmons et al., 2017). Conflicting results on this topic were showm by Lu et al. (2016), where despite confirmation that RBPJ directly binds to the YAP promoter, this action suppressed rather than stimulated YAP transcription. As a result, Notch signaling was found to promote the differentiation of the hepatic stem cells and simultaneously inhibiting their proliferation through the reduction of YAP activity (Lu et al., 2016). Other studies further highlight the complexity of the Notch and YAP/TAZ crosstalk within a single tissue. For example, one study showed that YAP positively upregulated Notch receptor Notch1 in hepatocytes which also revealed a novel YAP/TAZ-Notch1-NCID axis in hepatocytes and liver regeneration (Liu et al., 2019). Further, inhibition of YAP/TAZ signaling *in vivo* confirmed the impact of inhibiting this signaling pathway on liver regeneration. Additionally, the findings of this group indicated that inhibition of the YAP/TAZ signaling pathway resulted in a decrease in the protein levels of molecules linked to the Notch signaling pathway. This indicated a regulatory function of the YAP/TAZ pathway in influencing the Notch signaling pathway during liver regeneration (Liu et al., 2019).

Another recent study demonstrated that Notch and YAP/TAZ simultaneously upregulate the function of each other. Wang et al. (2024) showed that YAP/TAZ activated through cell‐matrix interactions results in the transcriptional upregulation of Notch1 and Delta‐like ligand 4 (DLL4). These molecules, in turn, served as direct positive regulators of the phenotype of the type H endothelial cells (THECs) in bone marrow endothelial cells (BMECs) following induction of distraction osteogenesis using tensile stress (TS). Concurrently, the Notch intracellular domain increased the activity of YAP/TAZ by boosting the transcriptional upregulation of YAP and the stabilization of TAZ protein, thereby establishing a correlation between YAP/TAZ and Notch (Wang et al., 2024). Finally, it is plausible that the specific regulation of YAP/TAZ activity by Notch signaling could exhibit variability based on the distinct cell types, relying on intricate interactions with other signaling mechanisms inherent in diverse cell lineages (Heng et al., 2021). As both Notch and YAP/TAZ signaling play an inherently important role in brain development, it is plausible that cell-type dependent crosstalk may influence the impact of these signaling pathways on neurodevelopmental disorders and brain cancers.

### Wnt signaling

The Wingless (Wnt) signaling pathway is critical in regulating both embryonic development and tissue self-renewal (Liu et al., 2022; Steinhart and Angers, 2018). The onset of the β-catenin-dependent canonical Wnt signaling pathway occurs when the Wnt ligand binds to Frizzled (Frz) and the low-density lipoprotein receptor-related protein (LRP)5/6 coreceptors. In the absence of Wnt, β-catenin undergoes phosphorylation and is sequestered in the cytoplasm by a complex involving Axin, adenomatous polyposis (APC), glycogen synthase kinase 3 (GSK3), and casein kinase 1 (CK1). Phosphorylated β-catenin then interacts with the E3 ubiquitin ligase β-TrCP, leading to ubiquitination and subsequent proteasomal degradation (Stamos and Weis, 2013; Schaefer and Peifer, 2019; Konsavage et al., 2012). Upon Wnt stimulation, phosphorylation of LRP5/6 induced by Frz activates the scaffold protein Dishevelled (Dvl), which facilitates the recruitment of Axin to the receptors and inhibits β-catenin phosphorylation. The accumulated unphosphorylated β-catenin moves from the cytoplasm to the nucleus and promotes the transcription of Wnt target genes by interacting with T cell-specific factor (TCF)/lymphoid enhancer-binding factor (LEF) (Zhan et al., 2017; Clevers and Nusse, 2012). In the absence of Wnt stimulation, YAP and TAZ participate in the destruction complex, where they interact with Axin1. This complex acts as a cytoplasmic anchor and functional reservoir for YAP/TAZ. Upon exposure to Wnt ligands or loss of destruction complex components, such as Axin or APC, YAP/TAZ are rapidly released from the complex. This leads to their relocation to the nucleus and subsequent increase in YAP/TAZ/TEAD-dependent transcription. Thus, the activation of Wnt signaling displaces YAP/TAZ from the destruction complex, facilitating their nuclear accumulation and the initiation of target gene expression (Azzolin et al., 2014).

Studies have shown the activation of the Hippo pathway by the Wnt signaling pathway and *vice versa*, where YAP/TAZ regulate the Wnt pathway by modulating β-catenin activity (Li et al., 2019). In hepatocellular carcinoma (HCC), WNT7A inhibits the adipogenesis of fibro-adipogenic progenitors (FAPs) by inducing the nuclear translocation of YAP independent of β-catenin. Moreover, WNT7A promotes the nuclear retention of YAP and TAZ during FAP differentiation (Fu et al., 2023). Another study using intestinal organoids, showed that the modulation of Wnt signaling, either through Porcupine (Porc) inhibitor LGK974 or Wnt activation in APC homozygous mutants, results in changes in YAP mRNA and protein levels. It was also recognized that Wnt signaling governs the transcriptional control of YAP and TEAD genes. In contrast, the subcellular localization of YAP in intestinal organoids specifically involves Src family kinase signaling independently of Wnt pathways (Guillermin et al., 2021). In colon cancer cells, the levels of β-catenin and YAP proteins increase upon stimulation with WNT3A, implying that YAP may be a downstream target of the Wnt signaling cascade (Park and Jeong, 2015). A study showed earlier that nuclear β-catenin/TCF (T-cell factor) complexes bind to a DNA enhancer element within the first intron of the YAP gene. Consequently, the decrease of YAP mRNA and protein levels in colon cancer cells is attained through the suppression of β-catenin expression (Konsavage et al., 2012). Furthermore, WNT3A has been found to stabilize TAZ by preventing its interaction with 14-3-3 proteins through PP1A. This stabilization brings about the dephosphorylation of TAZ, its migration into the nucleus, and subsequent increase in activity (Byun et al., 2014). Extending on these findings, another group demonstrated that WNT3A and WNT5A/B enhance YAP/TAZ activity, but this activation occurs via an alternative pathway independent of the canonical Wnt/β-catenin signaling pathway, through the Wnt-FZD/ROR-Gα12/13-Rho GTPases-LATS1/2 axis (Park et al., 2015). Conversely, some studies have also shown the activation of Wnt signaling pathway by YAP/TAZ. Rosenbluh et al. (2012) and Wang Y. et al. (2017) showed that YAP is required for β-catenin dependent tumorigenicity via regulating its expression level, subcellular localization and transcriptional activity of β-catenin. YAP-overexpressing mice exhibit elevated expression of β-catenin and target genes (Lgr5 and Cyclin D), along with increased proliferation of intestinal epithelial cells compared to wild-type mice. Similarly, YAP knockdown has been shown to reduce β-catenin activation and downstream gene expression in both intestinal and gastric cells (Pan et al., 2017).

The TEAD family of transcription factors is crucial for the downstream genes induced by oncogenic YAP in the nucleus. Likewise, the activation of β-catenin-induced target genes relies on TCF/LEF family factors. Mechanistically, YAP can directly interact with β-catenin in the nucleus, forming a YAP/β-catenin/TCF transcriptional complex in cancer cells (Deng et al., 2018). Another investigation has revealed a novel mechanism by which YAP/TAZ regulates the Wnt/β-catenin signaling pathway, distinct from these studies. In this case, the investigators showed that the phosphorylation of YAP at Ser127 decreases the transcriptional activity of β-catenin/TCF and the subsequent gene expression by directly interacting with β-catenin. As a result, the Hippo pathway may inhibit the Wnt/β-catenin signaling pathway by impeding the nuclear translocation of β-catenin rather than by regulating its stability (Imajo et al., 2012). The crosstalk between Hippo-YAP/TAZ and β-catenin signaling also occurs during brain development, in neurodegenerative disorders and in glioma pathogenesis (Ouyang et al., 2020; Sileo et al., 2022).

## YAP/TAZ and the microenvironment

Cells possess remarkable capabilities for physical interaction with adjacent cells and their surroundings. They can perceive and react to mechanical stimuli by translating them into biochemical signals through a process termed mechanotransduction (Saraswathibhatla et al., 2023). The perception of mechanical cues, denoting physical forces applied to cells, is predominantly sensed by transmembrane proteins and the actin cytoskeleton. This regulatory mechanism entails various cellular components such as the cytoskeleton, the nucleoskeleton, integrins, and focal adhesions (FAs). These components trigger a sequence of intracellular processes, such as the activation of signaling pathways, ion channels, and transcriptional regulators (Ritsvall and Albinsson, 2024). Upon detection of force, cells respond by producing opposing forces of equal magnitude through the modulation of myosin motor activity to maintain equilibrium in the actin cytoskeleton (Janmey and Miller, 2011; Parsons et al., 2010). External mechanical forces can induce active modifications in the actin cytoskeleton. For instance, cellular stretching can result in continuous activation of RhoA and myosin, consequently leading to the generation of stress fibers (Torsoni et al., 2005; Liu et al., 2007; Cai and Sheetz, 2009). Several investigations have highlighted the functions of YAP and TAZ as mechanotransducers, exerting a dynamic impact on cellular characteristics like differentiation and the development of diseases (Lin et al., 2024). Initially documented by Dupont et al. (2011), it was proposed that the activation of YAP and TAZ occurs independently of the Hippo pathway in response to diverse mechanical stimuli, such as extracellular matrix stiffness, cellular geometry, and cytoskeletal tension. The authors also showed compelling evidence implying that the state of the F-actin cytoskeleton and the function of Rho GTPase are crucial for the regulation of YAP/TAZ under these conditions. Additionally, it was demonstrated that this regulatory mechanism could operate without the involvement of LATS, representing a non-canonical Hippo signaling pathway as discussed earlier (Dupont et al., 2011).

The extensive variety in cellular geometries reflects the diverse array of cellular morphologies that emerge throughout morphogenesis, remodeling, and planar polarization of tissues and organs. It is widely recognized that alterations in cell geometry can effectively regulate cell proliferation, a process that is monitored by YAP/TAZ in response to such changes (Lindsey et al., 2015; Gibson and Gibson, 2009). A research study replicating this result illustrated that YAP primarily localizes in the cytoplasm of cells occupying a small surface area, whereas it is distinctly concentrated in the nucleus of cells spread over a larger surface area (Wada et al., 2011). Another study showed a substantial change in the intracellular localization of YAP/TAZ proteins between soft and rigid matrices, with most cells showing predominant nuclear localization of YAP/TAZ on rigid matrices and a lesser number displaying distinct nuclear localization on soft matrices in idiopathic pulmonary fibrosis (Liu et al., 2015). A recent study also established that the stiffening of the extracellular matrix triggers aberrant activation of YAP/TAZ and reorganization of the cytoskeleton in Schlemm’s canal cells in the eyes, a process that can be completely reversed through matrix softening in a time-dependent manner (Li et al., 2024).

Mechanosensitive molecules situated in the cellular membrane play a vital role in sensing external mechanical cues, thereby initiating mechanotransduction. These molecular structures include integrins and focal adhesions. Integrins are known to regulate YAP/TAZ via focal adhesion sites which serve as an intermediary and are essential for transmitting such signals (Lin et al., 2024). Integrins respond to various extracellular molecules, such as collagen, laminin, and fibronectin (Humphries et al., 2006). When exposed to a rigid surface or high mechanical tension, integrins are activated and aggregated, causing structural alterations in components of focal adhesions. Consequently, downstream signaling molecules are recruited, initiating the organization of the actin-myosin cytoskeleton (Goldmann, 2012). For instance, in endothelial cells (ECs), signaling pathways mediated by the cell junction protein AmotL2, focal adhesions, and the nuclear lamina are essential for the transcription of YAP. A recent study demonstrated that mechanical forces detected at cell-cell junctions through AmotL2 directly impact global chromatin accessibility and the activity of EZH2, thereby influencing YAP promoter activity (Mannion et al., 2024). Furthermore, the application of mechanical force induces an allosteric effect in the extracellular region of integrin αVβ3, leading to increased levels of integrin αVβ3 and fibronectin. Consequently, focal adhesions aggregate and modulate the function of the actin cytoskeleton via downstream signals like RhoA GTPases, ultimately promoting F-actin assembly and YAP expression (Puklin-Faucher and Sheetz, 2009). A recent study illustrated that vinculin, a crucial protein in focal adhesions, governs the ECM stiffness-dependent localization of YAP/TAZ and enhances its nuclear translocation on rigid substrates. The study also revealed that vinculin does not affect the Hippo pathway since LATS1 levels remained consistent between control cells and cells lacking vinculin. Instead, vinculin interacts with F-actin, influencing its arrangement. In addition, studies using treatment with cytochalasin D, an actin polymerization inhibitor, propose that vinculin-mediated increase of YAP/TAZ nuclear localization and activity is connected to actin structure (Kuroda et al., 2017).

In summary, YAP/TAZ is a key regulator of cellular behavior influenced by the microenvironment, including the cytoskeleton, focal adhesions (FA), and integrins. The cytoskeleton’s dynamics affect YAP activity by transmitting mechanical signals through focal adhesions, where integrins play a critical role in sensing and responding to extracellular matrix stiffness. This interaction influences YAP localization and activity, ultimately impacting cell proliferation, differentiation, and survival. In recent years, mechanotransduction and the role of YAP/TAZ in mediating the response of cells to mechanical forces have been an area of intense investigation, especially in cancer cells that respond to mechanical changes in the tumor microenvironment (Piccolo et al., 2023; Panciera et al., 2017; Di et al., 2023). However, much less is known about the transduction of mechanosensitive signals in neurons in response to mechanical changes in the brain extracellular matrix.

## Role of YAP/TAZ in brain development

The human brain is the most complex structure known as evidenced by its myriad neuronal and non-neuronal cells and the trillions of cellular connections, enabling the generation of a wide array of cognitive and behavioral responses (Sahu and Mondal, 2021). For the proper development of our intricate nervous system, it is essential to ensure precise spatial and temporal regulation of various signaling pathways. During brain development and maturation, the Hippo pathway has been continuously implicated (Terry and Kim, 2022). Numerous research works have highlighted the involvement of YAP/TAZ in the generation of diverse brain cells. These investigations showcase the ability of this pair to connect numerous biological processes to transcriptional output, serving as an effective mechanism to harmonize multiple cellular and molecular processes during and after development (Terry and Kim, 2022). Mammalian brain development starts with the formation of the neural tube and progresses to the development and refinement of the cortex, cerebellum, cerebrum and ventricular system (Terry and Kim, 2022; Sahu and Mondal, 2021; Lavado et al., 2021). This segment explores the conventional function of YAP/TAZ in the context of brain development (Table 1).

**TABLE 1:** Model systems used to evaluate YAP/TAZ signaling cascades in brain development, neurodevelopmental disorders, and cancer.

	Signaling molecules	Model system	Major findings	Ref.
BRAIN DEVELOPMENT AND NEURODEVELPMENTAL DISORDERS	AMOG-NF2-YAP/TAZ	Cerebellar granule cells	Actin cytoskeleton reorganization conducive for cerebellar development	Litan et al. (2019)
	YAP/TAZ	Deletion of YAP/TAZ using YAP^F/F^;Taz^F/F^ mice and Emx1-Cre	Deletion of YAP and TAZ in radial glial cells perturbs cortical development resulting in reduced numbers of cortical projection neurons and hydrocephaly	Lavado et al. (2021)
	YAP/TAZ-Fox genes	Ablation of YAP and TAZ in cranial neural crest by crossing Yap^flox/+^ and Taz^flox/+^ mice with Wnt1^Cre^ mice; O9-1 cells with YAP deletion	Loss of YAP and TAZ leads to abnormalities in neural tube closure and cerebellar aplasia similar to Dandy-Walker syndrome through regulation of Foxc1	Wang et al. (2016)
	MST1//2-LATS1/2-YAP/TAZ-TEAD	Overexpression of YAP and of transcriptionally active form of TEAD1 in the neural tube of chick embryos	Overexpression of YAP causes expansion of neural progenitor pool; YAP and TEAD loss of function leads to increased cell death; Hippo pathway regulates NPC proliferation and fate choice during neural tube development	Cao et al. (2008)
	TEAD2	TEAD2 knockout mice	Neural tube closure defect/exencephaly	Kaneko et al. (2007)
	LPA/N-cadherin-YAP/TAZ	*In utero* electroporation of pregnant mice with YAP mutants using Nestin Cre; LPA treatment	Severe hydrocephalus	Park et al. (2016)
	YAP/TAZ	Deletion of YAP/TAZ using YAP^F/F^;Taz^F/F^ mice using Nestin-Cre or Math1-Cre	YAP/TAZ are required for cerebellar morphogenesis and folia development by establishing the radial glia scaffold and cellular polarity in neural progenitors during embryogenesis	Hughes et al. (2020)
	DGL5-MST1//2-LATS1/2-YAP/TAZ	CNS-specific knockout of Dlg5 in mice using Nestin-Cre; CNS-specific deletion of Mst1/2 with Nestin-Cre; Taz^−/+^ and Yap^−/+^ mice	Connection between cell polarity proteins and Hippo pathway; brain phenotypes in DLG5 KO mice are due to decreased YAP/TEAD levels	Kwan et al. (2016)
	P13K-YAP/TAZ	Mice with tet-inducible activating Pik3ca^H1047R^ mutants in embryonic cortical progenitors	Abnormal PI3K-YAP interaction disrupts ependymal development in forebrain; hydrocephalus	Roy et al. (2019)
	LATS1/2-YAP/TAZ	Expression of YAP1/TAZ and nlsYAP5SA mutants and LATS1/2 conditional knockout mice using NEX^Cre/Cre^	Suppression of YAP1 activity in NEX/NeuroD6 expressing neuronal precursors is essential for neuronal differentiation whereas activation of YAP1 leads to ependymoma-like tumors	Eder et al. (2020)
	HIPPO-PARD3; NOTCH-YAP/TAZ	Conditional Pard3 knockout using Pard3^fl/fl^ with Emx1-Cre and Nex-Cre mice; YAP/TAZ conditional double knockout using Emx1-Cre	Loss of PARD3 in cortical radial glial progenitors causes severe malformations of the cortex and increased seizure susceptibility; effect is suppressed by simultaneous removal of YAP/TAZ	Liu et al. (2018)
	YAP-TEAD1-4	NIH/3T3 fibroblasts	Identification of YAP/TEAD protein binding domains	Vassilev et al. (2001)
	LATS1/2-YAP/TAZ	LATS1/2; p53 triple knockout mice with Emx1-Cre	YAP/TAZ activation resulting from Lats1/2 deletion leads to massive apoptosis of neural progenitors and global hypertranscription	Lavado et al. (2018)
	AMOT-YAP/TAZ-S6 KINASE	Deletion of Amot in neurons using Amot^fl/fl^ and Syn-Cre mice; Deletion of Yap1 in neurons using Yap1^fl/fl^ and Syn-Cre mice	Deletion of Amot in neurons reduced complexity of dendritic trees in hippocampal cells and cerebellar Purkinje cells through regulation of S6 kinase signaling and is independent of Hippo and TEAD; knockout mice present with impairment of motor coordination	Rojek et al. (2019)
	YAP/TAZ-ARHGAP18-CORTICAL ACTOMYOSIN NETWORK	Medaka and zebrafish mutant strains; morpholino knockdowns	Flattening of neural tube	Porazinski et al. (2015)
	GNAQ-YAP/TAZ; GNB2-YAP/TAZ	Skin biopsies of patients with Sturge-Weber Syndrome, cultured endothelial cells, keratinocytes and fibroblasts	Mutations in GNAQ and GNB2 reduce YAP levels in patients with the neurological disorder of Sturge-Weber Syndrome	Fjaer et al. (2021)
	TRPV4-Ca^2+^-YAP-STAT3	Mice with 4-AP (4-aminopyridine)-induced seizures	Astrocytic TRPV4 activation promotes upregulation and activation of YAP by increasing Ca^2+^, promoting the release of proinflammatory cytokines and neuroinflammation through STAT3	Zeng et al. (2022)
	GRM7-CREB-YAP/TAZ	GRM7 knockdown by *in utero* electroporation of pregnant mice	Grm7 knockdown affects neuronal development; GRM7 acts through CREB and YAP to regulate neurogenesis	Xia et al. (2015)
CANCER	EGFR-MOB1- YAP/TAZ-TEAD	Head and neck squamous carcinoma cell cultures (CAL33, CAL27, and HN6); tumor xenografts	Activated EGFR induces YAP/TAZ activation through phosphorylation of MOB1 and reduced LATS1/2 function thereby contributing to therapy resistance	Ando et al. (2021)
	YAP-IGF2-PI3K/Akt	Irradiated cerebellar neural precursors from neonatal mice; NeuroD2-SmoA1 mice; Patched^+/−^ mice; tumor xenografts	YAP expression permits cell proliferation and survival after radiation-induced DNA damage through induction of IGF2-expression followed by activation of the PI3K/Akt pathway	Fernandez et al. (2012)
	YAP/TAZ-SEPTIN10-CAPZA2 + MAP4-YAP/TAZ	Hepatocellular carcinoma cell lines (HepG2, HLF); human HCC (hepatocellular cancer) tissues	SEPTIN10 is a YAP/TAZ target gene that promotes HCC cell migration and invasion through modulating actin and microtubule networks with a feedback on YAP/TAZ activity	Weiler et al. (2024)
	MST1/2-LATS1/2-YAP/TAZ; MAP4Ks-NF2-LATS1/2-YAP/TAZ	Wwc1^F/F^, Wwc2^F/F^, Nf2^F/F^, Sav1^F/F^, Mst1^F/F^ and Mst2^Δ/Δ^ mice crossed with Alb-Cre mice	The MST1/2-SAV1-WWC1-3 (HPO1) and MAP4K1-7-NF2 (HPO2) Hippo signaling modules together regulate LATS1/2 kinases and YAP/TAZ; inactivation of either module in liver results in bile duct hyperplasia and intrahepatic cholangiocarcinoma	Qi et al. (2023)
	NF2-YAP/TAZ-PTGS2, COX2, AREG-EGFR	Intraneural injection of YAP reporter constructs into the sciatic nerves of NOD/SCID mice; NF2 cell lines (SC4; HEI-193, HSC2λ)	YAP function is required for NF2-null Schwann cell survival, proliferation, and tumor growth through target genes such as PTGS2, COX-2, and AREG (amphiregulin)	Guerrant et al. (2016)
	AXIN1-YAP/TAZ	Hydrodynamic tail vein injection of c-Met/β-catenin/Cre plasmids in Yap^flox/flox^;Taz^flox/flox^ mice	Axin1 binds to YAP/TAZ to regulate YAP/TAZ stability; activation of Hippo via overexpression of Lats2 or deletion of YAP/TAZ significantly inhibits c-Met/sgAxin1-driven HCC development	Liang et al. (2023a)
	INTEGRIN-FAK-SRC-YAP/TAZ	Chemically induced colitis-associated colorectal tumor formation in Postn^−/−^ and Apc^Min/+^ mice; colorectal adenocarcinoma cell lines (CMT93, DLD1)	Cancer-associated fibroblast-derived periostin promotes colorectal tumorigenesis through integrin-FAK-Src-YAP/TAZ signaling	Ma et al. (2020)
	STAT3-YAP/TAZ	Metastatic colorectal cancer xenograft (CT26) models; Deletion of Yap/Taz in endothelial cells using Yap^lox/lox^Taz^lox/lox^ mice crossed with Cdh5-(PAC)-Cre^ERT2^ mice (tamoxifen-inducible); Stat3 deletion using Stat3^lox/lox^ crossed with Cdh5-(PAC)-Cre^ERT2 mice^	VEGF and TNFa promote the interaction of YAP/TAZ with STAT3 in endothelial cells in tumor angiogenesis; treatment with YAP/TAZ inhibitor verteporfin reduced tumor progression in mouse colorectal cancer	Shen et al. (2021)
	VASN-YAP/TAZ-TEAD-CTGF + PTEN/PI3K/AKT	Various cancer cell lines; patient colorectal cancer tissues	VASN interacts with YAP to activate YAP/TAZ-TEAD target genes CTGF and the PTEN/PI3K/AKT pathway to stimulate colorectal cancer cell proliferation	Liang et al. (2023b)
	YAP/TAZ-MCM7-miRs-25, 93, 106b-p21	Non-small cell lung cancer cell lines (H1299, H1975)	In lung cancer YAP/TAZ may regulate p21 protein abundance through different mechanisms including the p53-independent MCM7, miRs-25, 93, 106b mechanism	Lo Sardo et al. (2017)
	YAP/TAZ-NUAK2	Various cancer cell lines; tetracycline-inducible YAPS127A expressing mice (TetO-YAP-S127A); NUAK2/YAP knockdown in TetO-YAP:Cas9 mice	NUAK2 is a target of YAP and participates in a feedback loop to modulate YAP-driven hepatomegaly and liver cancer growth	Yuan et al. (2018)
	OTUD7B-YAP1-NUAK2	Tumor xenografts using cancer cells with OTUD7B knockdown	OTUD7B promotes gastric cancer progression by enhancing the activity of the YAP1/NUAK2 axis	Guo and Guo (2024)
	NUAK2-MST/MOB/LATS-YAP/TAZ-TEAD-NUAK2	NUAK2 knockout MDA-MB231 cells and orthotopic tumor xenografts	NUAK2-YAP/TAZ-NUAK2 is a feed forward loop that promotes tumorigenesis in diverse cancers	Gill et al. (2018)

Brain development initiates from the neural tube which is comprised of actively dividing neuroepithelial cells also recognized as neural progenitor cells (NPCs) that exhibit elevated levels of YAP (Wang et al., 2016; Milewski et al., 2004). The elimination of YAP from WNT1-expressing cells, encompassing cells located in the dorsal region of the neural tube, roof plate, and neural crest, has been demonstrated to lead to abnormalities in neural tube closure (Wang et al., 2016). Also, an earlier study showed that either the activation of YAP/TEAD or the suppression of MST1/2 and LATS1/2 can trigger the upregulation of Ccnd1 (cyclin D1), facilitating the NPC cycle process. Activation of YAP/TEAD can also diminish the expression of neurogenic b-HLH (basic helix-loop-helix) factor NeuroM, impeding NPCs’ differentiation. Conversely, inhibiting YAP/TEAD can instigate apoptosis in NPCs (Cao et al., 2008). It has been shown that the inability of the neural tube to close results in severe congenital malformations like anencephaly and spina bifida, characterized by the protrusion of CNS neural tissue from its usual domain (Avagliano et al., 2019). Evidence substantiating the involvement of YAP in neural tube closure is demonstrated by TEAD2 conditional knockout mice exhibiting exencephaly, denoting brain tissue protrusion beyond the skull due to the failure in achieving anterior neuropore closure (Kaneko et al., 2007). In a recent investigation involving a family with consecutive fetuses displaying anencephaly, mutations in Nuak2, an upstream negative modulator of Hippo signaling, were identified to diminish YAP activity. It was suggested that this reduction in YAP activity could be the underlying cause of anencephaly (Bonnard et al., 2020). Cao et al.’s (Cao et al., 2008) studies on chick embryos have revealed that the regulation of YAP activity is crucial for the normal proliferation of neural progenitors and the prevention of premature differentiation of progenitor cells. The interaction between YAP and TEAD4 promotes the expression of CyclinD1. In this scenario, decreasing YAP levels resulted in elevated cell death and narrowing of the neural tube, whereas increasing YAP levels boosted precursor proliferation but ultimately reduced the quantity of neurons (Cao et al., 2008). Taking into consideration the above data, it can be concluded that YAP plays a key role in normal neural progenitor cells proliferation and differentiation during early brain development stage and its dysregulation can affect neural tube formation.

The significance of YAP in brain development is also apparent in ependymal cells and choroid plexus epithelial cells found in the ventricular system. After the reduction of neural progenitors in the ventricular zone (VG), a stratum of multiciliate ependymal cells develops to sheathe the ventricles. These ciliated cells move cerebrospinal fluid (CSF) through the ventricular system, which contains various soluble factors that influence NSC (neural stem cell) lineage and cell survival during development (Deng et al., 2023; Nelles and Hazrati, 2022). It is noteworthy that YAP/TAZ are prominently expressed in choroid plexus cells (Park et al., 2016; Hughes et al., 2020), which are involved in CSF production. This development begins around embryonic day 12.5 (E12.5) in mice and at 6 weeks post-conception in humans (Lun et al., 2015). Despite their significant levels, the functions and developmental roles of YAP/TAZ in the choroid plexus are not well understood and require further research. YAP expression persists in ependymal cells throughout adulthood, and its absence has been linked to structural defects in the ventricular system (Park et al., 2016). In nervous system-specific YAP mutants generated using Nestin-Cre, severe hydrocephalus is a prominent phenotype characterized by CSF accumulation and ventricular enlargement due to disrupted aqueduct integrity (Park et al., 2016). One factor contributing to aqueduct stenosis development is the insufficient presence or developmental failure of ependymal cells, which compromises the aqueduct ventricular wall integrity and obstructs CSF flow. Dysregulated YAP signaling is also associated with hydrocephalus pathogenesis through different causes, such as Dlg5 mutants and posthemorrhagic hydrocephalus induced by lysophosphatidic acid (LPA) injection. Dlg5 conditional knockout animals (Nestin-Cre) with reduced YAP/TAZ levels exhibit impaired ependymal cell development. This effect was reversed by simultaneous MST1/2 conditional knockout, leading to ependymal cell layer restoration and aqueduct patency (Kwan et al., 2016). In LPA-induced hydrocephalus, YAP reduction by LPA treatment caused detachment of ventricular lining cells, hindering ependymal cell development and resulting in aqueduct stenosis (Park et al., 2016). While LPA reduces YAP levels in neural progenitors and ependymal cell precursors, YAP overexpression before LPA injection can restore junctions and ventricular attachments, highlighting the significant role of YAP loss in LPA-induced cellular disruption. Conversely, YAP hyperactivation can also impact ependymal cell development, with PI3K overactivation in hGfap-Cre mice leading to abnormal positioning and overproduction of radial glia/ependymal cell precursors, associated with abnormal brain folding and enlarged ventricles (Roy et al., 2019). Moreover, YAP hyperactivity induced by LATS1/2 double-conditional knockout or YAP5SA expression through Nex-Cre results in an increased number of ependymal-like cells resembling ependymoma, a tumor of ependymal cells (Eder et al., 2020). Overall, this evidence suggests that the loss of YAP function reduces the number of ependymal cells, while overactivation of YAP leads to an excess of ependymal-like cells. Thus, the essential role of YAP in ependymal cell development is clearly demonstrated.

During cortical neurogenesis, which starts approximately at 7 weeks post-conception and peaks around 27 weeks post-conception in humans, neurons are generated through rapid cell division of neural progenitors, followed by gliogenesis (Silbereis et al., 2016). There are two distinct categories of progenitors present during cortical neurogenesis: multipotent apical neural progenitors and lineage-restricted progenitors situated at the base. The initial neuroepithelial cells transition into elongated apical radial glial (aRG) cells, also called ventricular radial glial (vRG) cells. Then the basal progenitors, specifically neurogenic intermediate progenitor cells (IPCs), divide in the subventricular zone with basal radial glia (bRG) also known as outer radial glia (oRG) dividing in the outer subventricular zone (Savini et al., 2019). Numerous research works have reported elevated levels of YAP in the developing cortices of mice and humans. Upon gene expression analysis of the developing mouse cortex, subsequent separation into distinct cell types reveals a high expression of YAP and TAZ in aRGs (Mukhtar et al., 2020). Investigations on YAP/TAZ double-conditional knockout animals have confirmed the essential role of YAP/TAZ in the generation of cortical neurons (Lavado et al., 2021; Liu et al., 2018). The decline in cortical neuron production due to YAP/TAZ loss is believed to be partially linked to a decrease in the number of aRG. While one study did not detect alterations in aRG density at E14.5 (Liu et al., 2018), Terry and Kim (2022) indicated that deletion of YAP/TAZ reduced the aRG population and the proportion of proliferating aRG at E16.5 by reducing cell cycle reentry and extending the cell cycle. Notably, the decline in proliferating aRG does not lead to an extreme production of IPCs or early born neurons; instead, there is a decrease in the quantity of IPCs, early born neurons, and late-born neurons. This highlights the necessity of YAP/TAZ not only for aRG proliferation but also for the subsequent generation of aRG-derived cells (Terry and Kim, 2022). Studies involving YAP deletion with Nestin-Cre (Park et al., 2016; Huang et al., 2016) or Emx1-Cre (Shao et al., 2020) did not observe proliferation defects at E14.5; however, a recent examination with Nestin-Cre at E15.5 showed a decline in cells in mitosis and S-phase, indicating reduced proliferating aRG. The differences in results might be due to the timing of these studies (E14.5 vs. E 15.5) which suggests that changes in the proliferating cell fraction may be noticeable at later time points. Nevertheless, since the number of aRG remained unaltered in YAP single mutants unlike YAP/TAZ double mutants (Lavado et al., 2021), the impact of reduced aRG proliferation on the aRG population remains unclear. The consequences of YAP loss alone on IPC number and neuron production are not definitively elucidated. Like YAP/TAZ double-conditional knockout studies, YAP single conditional knockout with Nestin-Cre displayed a decrease in IPCs, and uniquely reduced the number of late-born neurons, not early born neurons, at P0 (Vassilev et al., 2001). Conversely, no changes in neuronal production were observed at P21 when YAP was exclusively deleted with Emx1-Cre (Shao et al., 2020). These inconsistencies could stem from variations in the Cre lines utilized or the different time points evaluated, given that cortical development is still in progress at P0 (when Nestin-Cre mice were examined) but not at P21 (when Emx1-Cre mice were studied). In gain of function studies, overexpression of unaltered YAP or the expression of mutated YAP constructs resulted in an increase in the aRG population and a decrease in the production of IPCs and neurons, indicating YAP’s role in impeding aRG progression towards a lineage-specific or specialized state. It is worthy of note that activation of YAP has been successfully accomplished by the overexpression of wild-type YAP or by utilizing various YAP mutant constructs that contain sites resistant to LATS1/2 phosphorylation. These sites involve specific amino acid substitutions, such as serine to alanine at positions 127 (1SA), 127 and 381 (2SA), and 61, 109, 127, 164, 381 (5SA), which have been employed in numerous *in utero* electroporation experiments. The most severe consequence was noted when the YAP5SA mutant structure is expressed, leading to potential cell death (Lavado et al., 2018). The impacts of increased YAP5SA levels are somewhat mitigated by the presence of YAPS94A, which eliminated the interaction between YAP and TEADs, implying that the gene transcription regulated by TEADs contributes to the repercussions of excessive YAP5SA. In a different investigation, cells overexpressing YAP5SA formed clusters proximal to the lateral ventricle and sparked non-cell autonomous gliogenesis (Han et al., 2015). Conversely, in another analysis, the positioning of YAP5SA-expressing cells was altered towards the vicinity of the cortical ventricular (VZ) and cortical subventricular (SVZ) regions (Cappello et al., 2013). These gain-of-function investigations corroborate the notion that maintaining optimal levels of YAP/TAZ is vital for typical cortical development in the brain.

Cerebellar development is characterized by a prolonged duration and results in the generation of the most abundant neurons in the human brain, namely, the cerebellar granule neurons (Cho et al., 2011). This intricate process is highly susceptible to environmental insults during birth, as well as cerebellar injuries resulting from premature birth, often leading to reduced cerebellar size (Steggerda et al., 2009). Recent genetic investigations have unveiled the critical involvement of YAP/TAZ in cerebellar development and the recuperation following early postnatal injuries. A recent study has established a functional association between the Na, K-ATPase β2 subunit/adhesion molecule on glia (AMOG) and the Hippo pathway activator Nf2 (Neurofibromin-2/Merlin) during the differentiation of cerebellar neurons. In cerebellar granule precursor cells, AMOG exerts negative regulation on the expression of Nf2, thereby enhancing YAP activity and facilitating actin cytoskeleton reorganization conducive to cerebellar development (Litan et al., 2019). Further substantiating the expression and function of YAP in cerebellar neurons is evidence indicating that the absence of YAP in neurons results in morphological abnormalities in Purkinje cell dendrites, characterized by reduced dendritic complexity and motor coordination deficiencies. YAP Syn-Cre conditional knockout animals exhibit an increased number of granule neurons within the molecular layer, implying impaired migration in YAP-deficient granule neurons. These findings collectively suggest that YAP displays dynamic expression in various cell types during and after cerebellar development, with its depletion leading to discernible functional deficits. YAP/TAZ double-conditional knockout (Nestin-Cre) animals display diminished cerebellar size (Hughes et al., 2020; Rojek et al., 2019) and a flattened embryonic cerebellar shape, resembling the flattened morphology observed in medaka fish embryos carrying a mutated form of YAP (Porazinski et al., 2015). Despite investigations into the role of YAP/TAZ in granule cell progenitor (GCP) proliferation, conflicting evidence has emerged from studies in cell culture and genetic studies. While YAP overexpression has been linked to increased GCP proliferation in cell culture, YAP knockdown has been associated with decreased GCP proliferation (Rojek et al., 2019; Fernandez et al., 2009). Genetic deletion of YAP in GCPs using Nestin-Cre did not impact the GCP proliferation rate or folia formation. Additionally, YAP/TAZ deletion did not hinder GCP over-proliferation induced by SmoM2, an activated allele of Smoothened (Yuan et al., 2022), indicating that the *in vivo* function of YAP/TAZ in GCP proliferation may not be essential for normal physiological functioning or in the context of type II medulloblastoma mediated by SHH signaling. It is of interest to note that YAP plays a crucial role in the restoration of cerebellar size and architecture after radiation-induced damage during the early postnatal period. The absence of YAP in these precursor cells within the cerebellum diminished the capacity of the cerebellum to undergo regeneration post irradiation. Despite the normal initial proliferation of regenerated GCPs, the survival of cells was notably compromised in the absence of YAP. Intriguingly, the simultaneous removal of both YAP and TAZ did not impact the recovery of GCPs, despite the necessity of YAP for the survival of regenerated GCPs, indicating a potentially intricate interaction between these two paralogs in the process of regeneration post injury (Yang and Joyner, 2019).

## A role for YAP/TAZ in neurodevelopmental disorders

Neurodevelopmental disorders (NDDs) are defined by the inability to attain cognitive, emotional, and motor developmental milestones. Typically, NDDs are linked with the disruption of the intricately synchronized occurrences that facilitate brain maturation. Conditions such as autism spectrum disorder (ASD), intellectual disability (ID), attention deficit hyperactivity disorder, and epilepsy fall within the spectrum of NDDs (Parenti et al., 2020). While research endeavors have sought to establish a connection between the etiology of neurodevelopmental disorders and the Hippo-YAP/TAZ pathway (Table 1), a direct implication of YAP/TAZ in most neurodevelopmental disorders remains elusive.

Sturge–Weber syndrome is identified as a neurocutaneous disorder distinguished by vascular malformations impacting the skin, eyes, and leptomeninges of the brain, which can give rise to conditions such as glaucoma, seizures, and intellectual disability. Seizures represent an epileptic manifestation of Sturge-Weber syndrome, and the pathological transformations induced by this syndrome contribute to the hyperactivation of the MAPK pathway (Comi, 2015; Fjaer et al., 2021). The gene GNAQ encodes a G-protein α-subunit (Gαq) of heterotrimeric G-proteins, and the mutation associated with Sturge–Weber syndrome manifests as an activating mutation, prompting an elevation in downstream pathways including MAPK and YAP. Interestingly, in patients negative for the GNAQ mutation, a novel somatic mutation in GNB2 was unveiled, encoding a β-subunit of the heterotrimeric G-protein complex. Notably, this mutation presents an alternative molecular groundwork for mutated G-protein signaling in Sturge–Weber syndrome. The expression of mutant and wild-type GNAQ and GNB2 instigated distinct MAPK phosphorylation levels, while inducing analogous alterations in the YAP pathway. This implies that the YAP pathway might hold greater significance in the pathogenesis of Sturge–Weber syndrome compared to the MAPK pathway (Fjaer et al., 2021). Conversely, a study has demonstrated that the absence of partitioning-defective 3 (PARD3) prompts radial glial progenitor cells (RGP) to undergo excessive neurogenesis, ultimately culminating in cortical augmentation and epilepsy. Furthermore, the inhibition of transcriptional co-activators YAP and TAZ within the Hippo pathway curtails excessive neurogenesis in RGP and diminishes seizure frequency, indirectly indicating a plausible association between epilepsy and the Hippo pathway, along with the involvement of YAP/TAZ (Liu et al., 2018).

A recent and different study examined the brains of 4-AP (aminopyridine)-induced mice and noted the activation and upregulation of TRPV4 (Transient Receptor Potential Vanilloid 4) in astrocytes, leading to increased [Ca2+] levels, enhanced nuclear translocation of YAP, elevated p-STAT3 levels, and subsequent upregulation of proinflammatory cytokines. The surge in A2 astrocytes intensifies neuroinflammation, causing disruptions in the brain’s microenvironment, thereby exacerbating seizure severity and neuronal impairment. Inhibition of TRPV4 chemically rebalanced the intracerebral immune microenvironment in mice, shielding neurons from extensive damage and reducing seizure severity. Additionally, the study implicated YAP in stimulating astrocyte activation and highlighted the involvement of the STAT3 pathway in fostering the release of proinflammatory cytokines in 4-AP-induced mice (Zeng et al., 2022). One more study has established an association between the metabotropic glutamate receptor 7 (GRM7) and brain developmental disorders such as attention deficit hyperactivity disorder (ADHD) and autism. GRM7 plays a role in neurogenesis by modulating the signaling pathways of CREB and YAP. The regulatory function of GRM7 in the proliferation and differentiation of neural progenitors is facilitated by its impact on the phosphorylation of CREB at Ser133 and the levels of YAP expression. Depletion of GRM7 function resulted in escalated phosphorylation of CREB at Ser133 (active form) and increased expression of YAP (active form). They also observed that the downregulation of GRM7 led to an upregulation in the expression of CyclinD1; a similar outcome was noted with the overexpression of YAP. The rise in the active YAP level resulting from the silencing of GRM7 subsequently triggered the upregulation of CyclinD1, which in turn facilitated the proliferation of NPCs; these occurrences align with the established role of YAP. Together this shows that GRM7 plays a role in regulating neurogenesis, at least in part mediated through YAP (Xia et al., 2015).

## YAP/TAZ in cancer development

As discussed earlier on, dysregulation of the Hippo pathway is a common occurrence in numerous human tumors, with YAP/TAZ activation being an indispensable hallmark for multiple cancer hallmarks. Within this segment, we discuss the role of the Hippo pathway in different types of human cancer (Table 1). A prominent function of YAP/TAZ in brain cancer is notably evident in high-grade gliomas, where they play a role in the advancement and progression of tumors, correlating with an unfavorable prognosis. Elevated levels of YAP/TAZ mRNA and protein are significantly identified in glioma tissues alongside their target genes, namely cysteine-rich angiogenic inducer 61 (CYR61), CTGF, and baculoviral IAP repeat-containing 5 (BIRC5) (Zhang H. et al., 2016). A recent investigation unveiled a non-transcriptional regulation of YAP/TAZ signaling in glioblastoma multiforme (GBM), pinpointing IMP1 as one of the highly expressed RNA binding proteins (RBPs) in mesenchymal GBM and glioma stem‐like cells (GSCs). IMP1’s recognition and binding to m6A‐modified YAP mRNA led to its stabilization and translation, consequently activating Hippo signaling. Furthermore, the study established that IMP1 establishes a feedforward loop with YAP/TAZ, thereby fostering GBM/GSC tumorigenesis and malignant advancement (Yang et al., 2023). The improper activation of YAP/TAZ in gliomas can be linked to LATS1/2 downregulation, instigating cancer progression (Zhang H. et al., 2016). Furthermore, chromobox homologue 7 (CBX7), a constituent of polycomb repressive complex 1 (PRC1) known as a YAP/TAZ suppressor, has been observed to be decreased in GBM because of promoter hypermethylation (Nawaz et al., 2016). Furthermore, YAP/TAZ can be stabilized by actin-like 6A (ACTL6A), which experiences upregulation in gliomas. The interaction of ACTL6A with YAP/TAZ disrupts the association with YAP-stem cell factor-beta-transducing repeat-containing E3 ubiquitin protein ligase (YAP-SCF-β-TrCP E3 ubiquitin ligase), thereby impeding YAP protein degradation (Ji et al., 2018).

Medulloblastoma is the most prevalent malignant pediatric brain cancer and originates from the cerebellum. Surprisingly, it is still not clear how YAP/TAZ promotes the development of this cancer, but earlier it was shown that YAP facilitates the acceleration of tumor growth and promotes radio-resistance in medulloblastoma, thereby fostering continuous proliferation following radiation exposure. The functionality of YAP allows cells to progress into mitosis despite DNA damage remaining unrepaired, achieved by inducing the expression of IGF2 and activating Akt, consequently leading to the deactivation of ATM/Chk2 and the circumvention of cell cycle checkpoints (Fernandez et al., 2012). More recently, a study demonstrated that the heightened activation of the hedgehog signaling pathway, which triggers YAP, results in the development of medulloblastoma within cerebellar granule neuron precursors. The nuclear localization of YAP has been observed across all histological subtypes of medulloblastoma, with desmoplastic nodular medulloblastomas displaying the most intense YAP immunopositivity (Ahmed et al., 2017). It remains to be determined whether YAP activation will be more prevalent in the distinct molecular subgroups of these tumors.

One of the cancers in which the role of YAP/TAZ is better defined is hepatocellular carcinoma (HCC), the most prevalent primary liver tumor with over 90% of cases (Asafo-Agyei and Samant, 2024). Numerous studies have shown that the dysregulation of Hippo signaling within the liver has the potential to induce significant hepatomegaly promptly, with YAP/TAZ playing an essential role in liver regeneration post-hepatectomy (Driskill and Pan, 2021).

A recent study showed that elevated expression of SEPTIN10 has shown a positive correlation with the dissemination of tumor cells, particularly evident through increased vascular invasion in HCC. SEPTIN10, identified as a direct target gene of YAP/TAZ, facilitates intracellular tension by modulating actin stress fiber formation. Additionally, its depletion has been noted to decrease the expression of established YAP/TAZ target genes at both mRNA and protein levels (Weiler et al., 2024). Frequent reduction of Succinate dehydrogenase enzyme (SDH) in samples obtained from HCC patients is associated with heightened succinate levels and poor prognoses within this patient population. Yuan et al. (2023) reported that the reduction of SDHA/B aids in the proliferation of HCC by impeding the proteasomal degradation of YAP/TAZ through the modulation of cullin1 NEDDylation. This connection ties SDH-deficient HCC cells to the YAP/TAZ pathway, rendering these cells susceptible to YAP/TAZ inhibition (Yuan et al., 2023). In murine models, the genetic inactivation of either the HPO1 or HPO2 module triggers a partial activation of YAP/TAZ, ultimately resulting in bile duct hyperplasia and the development of HCC. MST1/2-SAV1-WWC1-3 (HPO1) and MAP4K1-7-NF2 (HPO2), collectively regulate the activity of LATS1/2 kinases and YAP/TAZ co-activators transcriptionally. The inactivation of HPO1 uniformly activates YAP/TAZ, promoting cell proliferation throughout the liver and causing a proportionate and rapid increase in liver size. Conversely, the simultaneous inactivation of both HPO1 and HPO2 modules triggers a complete activation of YAP/TAZ, leading to the rapid onset of intrahepatic cholangiocarcinoma (iCCA) and premature mortality (Qi et al., 2023). Also, activation of Src family kinases has been observed to markedly increase tumor load in murine models by regulating the phosphorylation and nuclear translocation of YAP within human HCC (Guerrant et al., 2016). Another study has indicated that glycogen accumulation obstructs signal transduction pathways, subsequently inducing YAP activation, thereby contributing to liver enlargement and the progression of cancer (Liu et al., 2021). Lastly, YAP is identified as the primary effector of the Hippo pathway in c-Met/β-Catenin HCCs, with both YAP and TAZ being essential for c-Met/sgAxin1-dependent hepatocarcinogenesis. Mechanistically, AXIN1 interacts with YAP/TAZ in human HCC cells, influencing their stability. The genetic removal of YAP/TAZ has been shown to suppress pre-existing c-Met/sgAxin1 liver tumors, underscoring the crucial role of YAP/TAZ in tumor progression (Liang B. et al., 2023).

Colorectal cancer (CRC) arises from stem/progenitor cells located in the large intestine crypts responsible for maintaining intestinal equilibrium and enhancing regenerative capacity following intestinal injury (Thompson, 2020). One research study demonstrated the involvement of periostin, an extracellular matrix protein with multifunctional roles in inflammatory conditions and tumor spread (Ma et al., 2020). Periostin induces the activation of FAK-Src kinases through integrin-mediated signaling, resulting in YAP/TAZ activation and subsequent IL-6 expression in tumor cells. On the contrary, IL-6 initiates periostin expression in fibroblasts via STAT3 activation, thereby facilitating the progression of colorectal tumors. Another investigation revealed a correlation between the expression of YAP/TAZ and its downstream genes in endothelial cells (ECs) and tumor vascularization in human colorectal carcinomas. YAP/TAZ is known to induce genetic changes in ECs that support blood vessel formation, facilitating tumor growth. The study demonstrated that the cytokines VEGF and TNFα found in the tumor microenvironment enhance the interaction between YAP/TAZ and the transcription factor STAT3. The mechanism responsible for STAT3 nuclear import also facilitates the nuclear translocation of YAP/TAZ. The study also showed that treatment with the verteporfin, a YAP/TAZ small molecule inhibitor which functions by directly binding with the TEAD domain on the YAP protein, which disrupts the YAP/TAZ-TEAD interaction and consequently impeding the transcriptional activation of its downstream target genes crucial for tumor growth. This inhibitor reduced both vessel density and tumor advancement in a mouse model of colorectal cancer (CRC) (Shen et al., 2021). A recent study showed that Vasorin (VASN) can interact with YAP, thereby activating the YAP/TAZ and PTEN/PI3K/AKT pathways. VASN has been reported to be critical in tumor development and angiogenesis. The interaction between VASN and YAP inhibits YAP phosphorylation, promoting CRC cell proliferation, migration, and invasion through the activation of YAP/TAZ-TEAD target gene CTGF and PTEN/PI3K/AKT pathways. VASN, crucial in tumor growth and angiogenesis, displays upregulated expression in CRC compared to normal tissues. Additionally, the knockdown of YAP reversed the cellular changes induced by elevated VASN levels (Liang W. et al., 2023). Another study noted that HHEX (Haematopoietically expressed homeobox) forms associations with and stabilizes the YAP-TEAD complex on regulatory genomic loci, which, when induced by CK2, cooperatively regulates the expression of a subset of YAP/TEAD target genes that drive colorectal tumorigenesis. Casein kinase 2 (CK2) phosphorylates HHEX, enhancing its interaction with TEAD4 (Guo et al., 2022). Lastly, Wang Z. et al. (2017) demonstrated that ETAR (Endothelin receptor A) boosts colon cell proliferation, migration, and tumorigenesis by activating YAP/TAZ. ETAR stimulation operates through downstream G-protein Gαq/11 and Rho GTPase to inhibit the Hippo pathway, thereby activating YAP/TAZ, which is essential for ETAR-induced tumorigenesis (Wang Z. et al., 2017).

Lung cancer is one of the most frequently diagnosed malignancies and serves as the leading cause of cancer-related deaths (Thai et al., 2021). Among these, non-small cell lung cancer (NSCLC) is the predominant subtype (Lo Sardo et al., 2021). In the lung, the function of YAP/TAZ goes beyond regulating lung development to actively participating in the onset of lung cancer (Xie et al., 2018). Numerous studies have linked the upregulation of YAP/TAZ to tumorigenesis, disease progression, and unfavorable clinical outcomes in NSCLC (Su et al., 2012; Wang et al., 2010; Lorenzetto et al., 2014; Guo et al., 2017; Cui et al., 2012). A recent study demonstrated the interaction between YAP/TAZ and the SRCAP complex, leading to the promotion of oncogenic transcription and tumor growth in the lung. The SRCAP complex, a component of the INO80 subfamily, is a chromatin remodeling entity responsible for the replacement of canonical histone H2A with variant H2A Z in an ATP-dependent manner, thereby facilitating gene transcription (Zhang et al., 2024). ACTL6A, a known regulatory component of the ATP-dependent SWI/SNF chromatin-remodeling complexes, has been recognized as a key oncogenic driver in various types of tumors (Li et al., 2021). ACTL6A has been identified as a promoter of tumor growth and inhibitor of apoptosis in NSCLC through the Hippo/YAP signaling pathway. The upregulation of ACTL6A in NSCLC cells was correlated with increased proliferation and decreased rates of apoptosis. Conversely, the downregulation of ACTL6A led to growth inhibition and increased apoptosis in NSCLC cells (Ma and Shan, 2021). An earlier study demonstrated that YAP/TAZ activation triggers the transcription of the MCM7 gene and its hosted miRNAs (miR-25, miR-93 and miR-106b), ultimately fostering cell proliferation by inhibiting the post-transcriptional activity of the p21 cell cycle regulator. p21 levels were significantly reduced in lung tumors compared to healthy tissues but was restored through interference with YAP/TAZ or treatment with cerivastatin (Lo Sardo et al., 2017). A follow up study was done recently where the oncogenic miR-25, 93 and 106b binding the MCM7 gene were selected for further studies. The study revealed TGF-β Receptor 2 (TGFBR2) as a target of the miRNA cluster associated with significant prognostic implications due to its function as a tumor suppressor. In addition, the discovery was made that YAP/TAZ-mediated repression of TGFBR2 happens through post-transcriptional mechanisms involving the miR-106b-25 cluster, and through transcriptional regulation by interacting with the EZH2 epigenetic repressor. Furthermore, the study sheds light on the joint role of YAP/TAZ and EZH2 in the progression of lung cancer by collectively inhibiting a specific set of tumor suppressor genes, such as TGFBR2 (Lo Sardo et al., 2021). Numerous studies have investigated the regulation of the Hippo-YAP/TAZ pathway by various miRNAs and across different cancers. The findings of these studies have been summarized in several excellent reviews (Zhang et al., 2022; Sharma et al., 2022; Samji et al., 2021; Sadri et al., 2024; Ruiz-Manriquez et al., 2022; Lee et al., 2021); however detailed insights into specific regulatory mechanisms and their broader implications remain an area of ongoing exploration.

Wnt-YAP crosstalk was observed in ovarian cancer where the heightened expression of WISP2 (Wnt-inducible signaling pathway protein 2) was observed in various ovarian cancer tissues and cell lines. The absence of WISP2 hindered the proliferation, clonogenicity, and motility of ovarian cancer cells while facilitating apoptosis and influencing the cell cycle. This suppressive impact on growth, resulting from the removal of WISP2, was attributed to the suppression of p-ERK1/2, alongside CCAAT/enhancer-binding protein α (CEBPα) and CEPBβ. Furthermore, the lack of WISP2 resulted in the activation of the YAP protein (Shi et al., 2020). A separate investigation examining the function of Wnt-YAP in high-grade serous carcinoma analyzed the expression and clinical significance of Wnt pathway components. The mRNA levels of 20 molecules associated with Wnt signaling were identified in 87 high-grade serous carcinoma effusions. Subsequent analysis revealed the presence of YAP protein in 29 out of 34 (85%) high-grade serous carcinoma effusions and in all 18 surgical samples (Chehover et al., 2020) suggesting a link between Wnt and YAP signaling in these tumors.

Another YAP/TAZ regulatory axis, the canonical Hippo pathway involves MST1/2 and LATS1/2 which are integral upstream core kinase components of the Hippo pathway and serve as crucial tumor-suppressors. This has been implicated in different types of cancers (Yang et al., 2022). LATS1 expression is frequently reduced in gastric cancer tissues, and the absence of LATS1 expression is correlated with tumor invasion, poor prognosis, and recurrence in gastric cancer patients. Heightened LATS1 expression impedes cell proliferation and invasion both *in vitro* and *in vivo* by inhibiting the YAP signaling pathway (Zhang J. et al., 2016). In breast cancer, colorectal cancer, gastric cancer, and lung cancer, decreased LATS1/2 expression is associated with lymph node metastasis (Su et al., 2012; Cordenonsi et al., 2011; Liang et al., 2014). Recent research by Ando et al. (2021), demonstrated that EGFR activation in head and neck squamous cell carcinoma cells triggers the phosphorylation of the Hippo pathway component, MOB1, which inhibits LATS1/2 function, leading to YAP/TAZ activation. Furthermore, therapies targeting EGFR suppress YAP/TAZ, and the loss of LATS1/2-mediated YAP/TAZ activation contributes to therapy resistance (Ando et al., 2021). Another YAP/TAZ signaling axis that has been described in brain development is the YAP-NUAK signaling pathway. The Hippo-YAP pathway has been identified as a critical regulator in various cancers; nonetheless, the *in vivo* significance of YAP/TAZ target genes remains indeterminate. NUAK2 emerges as a crucial gene involved in the pathogenesis of diverse cancer types and represents one of the target genes regulated by the Hippo pathway. A study has demonstrated that NUAK2 contributes to a feedback mechanism that enhances YAP activity by stimulating actin polymerization and myosin function. Furthermore, the pharmacological inhibition of NUAK2 has been shown to repress YAP-dependent cancer cell proliferation and excessive liver growth (Yuan et al., 2018). A recent study investigation has spotlighted ovarian tumor domain-containing 7B (OTUD7B) as a deubiquitinase (DUB). OTUD7B overexpression has been linked to increased gastric cancer cell proliferation and metastasis both in experimental setups and in live subjects, while the suppression of OTUD7B has yielded contrary biological effects. Notably, OTUD7B has been discovered to enhance the activation of YAP1 by deubiquitinating and stabilizing it, thus resulting in an increase of NUAK2 expression. Through its actions, OTUD7B promotes the progression of gastric cancer by enhancing the activity of the YAP1/NUAK2 axis (Guo and Guo, 2024). Consequently, a subsequent investigation examined the expression patterns of NUAK2 in tissues through the development of a new NUAK2-specific monoclonal antibody, which was employed to distinguish NUAK2 expression profiles in normal skin and in 155 cases of various skin tumors including extramammary Paget’s disease (EMPD), squamous cell carcinoma (SCC), Bowen’s disease (BD), actinic keratosis (AK), basal cell carcinoma (BCC), and angiosarcoma (AS). Their analysis revealed that NUAK2 is frequently expressed in EMPD, SCC, BD, AK, BCC, and AS. They went ahead to investigate the expressions of YAP and p-Akt in these tumors. The expression p-Akt exhibited a positive correlation with tumor size in EMPD. Notably, the expression of NUAK2 showed a significant correlation with YAP in SCC (Al-Busani et al., 2020). NUAK2 expression levels are raised in prostate cancer and metastatic castration-resistant prostate cancer (mCRPC) relative to normal tissue, with elevated expression correlating with an increased risk of metastasis. Researchers observed that targeting NUAK2 *in vitro* resulted in reduced proliferation, diminished growth of three-dimensional tumor spheroids, and decreased Matrigel invasion by prostate cancer cells. Treatment with HTH-02-006 led to the inactivation of YAP and the reduction of NUAK2 and MYC protein levels (Fu W. et al., 2022). Lastly, a study pinpointed NUAK2 as a negative regulator of the Hippo pathway through a siRNA kinome screen, showing that NUAK2 aids in the nuclear localization of YAP/TAZ while functioning as a transcriptional target of YAP/TAZ, thereby forming a feed-forward loop that fosters tumorigenesis (Gill et al., 2018).

## The cerebellum - Highlighting the complexity of YAP/TAZ signaling in brain development, neurodevelopmental orders and cancer

In the previous sections, we discussed the role of YAP/TAZ in normal brain development, NDDs and various cancers. Most of the major signaling pathways that can modify YAP/TAZ activity in some ways have been implicated in both normal brain development as well as cancer progression, including brain cancers. These include the Hippo pathway, Shh and Notch signaling and receptor tyrosine kinase signaling cascades such as EGFR/ErbB and TGF-β signaling (Drakulic et al., 2022). The molecular details on how these major developmental pathways modulate YAP/TAZ activity within brain tumors are still an area of investigation and are even less defined in NDDs. Moreover, it is highly likely that these signaling pathways employ signaling networks and microenvironmental cues in a cell-type and tissue specific manner to drive diverse outcomes. While this adds a high level of complexity to the YAP/TAZ-associated signaling and transcriptional networks, it also provides opportunities for disease-specific targeting of YAP/TAZ in brain tumors and NDDs in drug development.

The cerebellum is one such example where cell-and tissue specific regulation of YAP signaling may contribute to both brain cancer and NDDs. The cerebellum, best known for its role in motor functions, has also been shown to participate in non-motor behaviors such as emotion, abstract thinking, and spatial navigation. Thus, injury or diseases affecting cerebellar circuitry can range from motor impairment to neurological disorders to neuropsychiatric conditions, including NDDs such as ASD, ADHD, and schizophrenia (Beckinghausen and Sillitoe, 2019; Sathyanesan et al., 2019; van der Heijden et al., 2021). Moreover, the most common malignant pediatric brain tumor, medulloblastoma, originates in the cerebellum (Northcott et al., 2019). Typical cerebellar development extends from early embryogenesis well into postnatal life until around 2 years of postnatal age in humans and 2 weeks postnatally in mice (Leto et al., 2016; Wizeman et al., 2019; Amore et al., 2021). Simplified, once the cerebellar anlage is established, the development of the cerebellar cortex is divided into four major phases - external granule layer expansion, granule cell migration, internal granule layer formation and Purkinje cell maturation - resulting in a rather well-defined cerebellar circuit. The cerebellar circuit receives two major excitatory inputs from mossy fibers and from climbing fibers. These inputs relay information to cerebellar granule cells whose axons ascend into the molecular layer where they bifurcate into parallel fibers and provide synaptic input into Purkinje cells. Purkinje cells are the only output cells of the cerebellar cortex and project to the deep cerebellar nuclei. Additional cells found in the cerebellum are modulatory neurons like basket, stellate and Golgi cells as well as glial cells such as radial glia, Bergman glia, oligodendrocytes and astrocytes. The intricate interplay of Purkinje cells with cerebellar granule cells and the migration of granule cells along radial glia and Bergman glia plays important roles in cerebellar corticogenesis, a process highly vulnerable to injury and environmental insults.

Recent studies have emphasized the function of YAP/TAZ in this intricate developmental process. Using genetic ablation experiments in mice, work by Hughes et al. (2020) demonstrated a role for YAP/TAZ in establishing secondary fissures during the normal developmental foliation of the cerebellum. Double conditional YAP/TAZ knockout mice presented with a small, flattened cerebellum and single knockout mice confirmed that only YAP and not TAZ played such a major role in cerebellar development. The authors further demonstrated that YAP or TAZ were not required for the proliferation of cerebellar granule cells, the most abundant cortical neurons in the cerebellum. Rather, radial glia fibers were misdirected and polarized cell architecture was disturbed leading to the developmental defects. Studies on the underlying mechanisms pointed to a disruption of junctional integrity and cell polarity in radial glia progenitors through improper localization of adherens junction proteins, apical polarity complex proteins and non-muscle myosin IIB (NMIIB) at the apical junction, all of which are required for cerebellar morphogenesis. Since Notch 1 and neuregulin-erbB signaling mediate the differentiation of radial glia when contacts between cerebellar neurons and glia cells are made (Patten et al., 2003), and mice with deletion of ErbB3 in radial glial and neuronal cells (GFAP-Cre) but not knockout mice with deletion of ErbB3 in granule cells (Math1-Cre) had aberrant cerebellar lamination (Sathyamurthy et al., 2015), it is tempting to speculate that these signaling pathways at least in part employ YAP in this process. The ErbB3 knockout mice also presented with impairments in balance and motor coordination but currently it is unknown whether the YAP/TAZ mutant mice displayed deficits in motor or social behavior mimicking NDDs. Moreover, it is important to note that in cerebellar development YAP signaling within the rather small population of radial glia regulates the proliferation of cerebellar granule cells that with over 90% are the most numerous neurons in the cerebellar cortex. In cancer, a similar theme emerges where YAP signaling in dysregulated normal cells such as immune or stromal cells within the tumor microenvironment can drive cancer cell proliferation and tumor growth. For example, Calvo et al. (2013) demonstrated a function of YAP in the tumor stroma where it was critical for the establishment and maintenance of cancer-associated fibroblasts (CAFs), which are known to have pro-tumorigenic functions. STAT3-YAP/TAZ signaling in endothelial cells promoted tumor angiogenesis enabling tumor expansion (Shen et al., 2021), highlighting the complexity of YAP/TAZ signaling. Targeting YAP/TAZ for therapeutic purposes in cancer and NDDs thus may need to expand beyond the traditional targets within tumor cells or neurons, respectively.

Cerebellar granule cells function as computational hubs that process signals incoming through mossy fibers and that integrate these signals through modulating synaptic plasticity at the interconnections of parallel fibers and Purkinje cells. Dysfunction of cerebellar granule cells has been associated with NDDs (Chen et al., 2022; van der Heijden and Sillitoe, 2021; Soda et al., 2019). On the other hand, the precursors of cerebellar granule cells are also the cells of origin of the Shh-subgroup of medulloblastoma, a malignant pediatric brain cancer (Northcott et al., 2019). As discussed in more detail in the above sections, YAP expressed in these cells has been implicated in the formation of normal cerebellar circuitry as well as medulloblastoma progression. Nevertheless, studies from conditional YAP/TAZ knockout mice and cultured tumor cells are somewhat conflicting. For example, while Hughes et al. (2020) suggested that YAP/TAZ have a minimal role in cerebellar tumorigenesis caused by constitutively activated Smo, in experiments using cell cultures, YAP overexpression was linked to increased GCP proliferation and YAP knockdown has been associated with decreased GCP proliferation (Rojek et al., 2019; Fernandez et al., 2009). Moreover, research into YAP function in cerebellar granule cells is further complicated in that they are the only excitatory cells in the cerebellum and functionally differ from other areas of the brain. With this comes a unique expression pattern of proteins, some of which have been shown to regulate Merlin/NF2 expression and with it YAP activity (Litan et al., 2019). Together, these studies point towards the need for appropriate experimental systems that are representative of cell type and tissue-specific microenvironmental factors to accurately identify cell-specific functions of YAP/TAZ. It should also be emphasized that both cancer and NDDs are multifactorial diseases where accompanying genomic changes and environmental factors can drive diverse outcomes (Figure 1).

## Conclusion

With our increasing understanding of genetic factors and mutations that contribute to NDDs such as ASD, ADHD, learning disorders and intellectual disability, it has become clear that the signaling pathways associated with these disorders significantly overlap with those dysregulated in cancer development and progression. While YAP/TAZ signaling has more recently emerged as one of the signaling pathways implicated in both cancer and NDDs, the most prominent examples where molecular mechanisms that promote cancer development/progression and NDDs intersect are the Ras/MAPK pathway and PI3K/mTOR signaling. For example, rasopathies are a group of genetic syndromes caused by germline mutations in the Ras/MAPK pathway. While unique in their own way, rasopathies share many common characteristics, including craniofacial dysmorphology, abnormalities in the musculoskeletal and ocular systems, cardiac malformations and not surprisingly, neurocognitive impairment and increased cancer risk (Rauen, 2013; Hebron et al., 2022). With the intense efforts that have been made to target Ras signaling for therapeutic purposes (Moore et al., 2020), we can take advantage of the knowledge that has been gained in this pursuit when targeting YAP/TAZ. Employing computational strategies such as machine learning and artifical intelligence (AI) to discover connections, similarities and differences of signaling pathways and networks while taking into account cell types, tissue microenvironment, developmental stages and genetic backgrounds (Nussinov et al., 2024), the emergence of tumoroids and novel organoid-based disease models for NDDs (Di Lullo and Kriegstein, 2017; Eichmuller and Knoblich, 2022) and new AI-based technologies in drug design and development (Qureshi et al., 2023) may be required when targeting a complex pathway such as YAP/TAZ signaling (Figure 1). Indeed, the fact that only a few tumor-promoting mutations have been reported so far, fusion proteins of YAP are more prevalent (Garcia et al., 2022) and YAP/TAZ tends to function as a hub to integrate extra-, inter- and intracellular oncogenic and tumorsuppressing signals, computational approaches will be particularly intriguing when developing novel therapeutics to modulate YAP/TAZ signaling.
